# Prospecting Plant Extracts and Bioactive Molecules with Antimicrobial Activity in Brazilian Biomes: A Review

**DOI:** 10.3390/antibiotics12030427

**Published:** 2023-02-21

**Authors:** José Carlos Eloi de Queiroz, José Roberto S. A. Leite, Andreanne Gomes Vasconcelos

**Affiliations:** 1 Department of Biomedicine, Centro Universitário do Distrito Federal, UDF, Federal District, Brasília 70390045, Brazil; 2 Research Center in Applied Morphology and Immunology, NuPMIA, Faculty of Medicine, University of Brasília, UnB, Federal District, Brasília 70910900, Brazil; 3 People&Science Pesquisa Desenvolvimento e Inovação Ltda, Federal District, Brasília 70790120, Brazil

**Keywords:** medicinal plant, infectious disease, antimicrobial

## Abstract

Antimicrobial resistance is currently one of the greatest threats to global health, food security, and development. In this aspect, medicinal plants have been studied to support the development of viable alternatives to prevent and treat infectious diseases. This study aimed to perform a review of the literature comprising the antimicrobial activity of vegetable species from Brazilian biomes. We selected 67 original scientific publications about extracts, fractions, or isolated molecules from plants in the Brazilian biomes, published between 2016 and 2020 in Pubmed, ScienceDirect, and Scielo. Data demonstrated that 98 plant species, especially collected in the Cerrado, Atlantic Forest, and Caatinga biomes, were tested against 40 fungi and 78 bacterial strains. Bioactive fractions of *Eucalyptus globulus* methanolic stump wood extract were active against *Candida albicans* and *C. tropicalis* (MIC 2.50 µg/mL). The catechin purified from *Banisteriopsis argyrophylla* leaves had activity against *C. glabrata* (MIC 2.83 µg/mL) and ethanolic extract obtained from *Caryocar coriaceum* bark and fruit pulp exhibited MIC of 4.1 µg/mL on *Microsporum canis.* For bacteria, compounds isolated from the dichloromethane extract of *Peritassa campestris*, lectin extracted from a saline extract of *Portulaca elatior* and essential oils of *Myrciaria pilosa* exhibited significant effect against *Bacillus megaterium* (MIC 0.78 µg/mL)*, Pseudomonas aeruginosa* (MIC 4.06 µg/mL) and *Staphylococcus aureus* strains (MIC 5.0 µg/mL), respectively. The findings support the antimicrobial and bioeconomic potential of plants from Brazilian biodiversity and their promising health applications.

## 1. Introduction

The number of bacteria and fungi presenting resistance against antimicrobial drugs in clinical use is increasing. According to the World Health Organization (WHO) [1], antimicrobial resistance is currently one of the greatest threats to global health, food safety, and development. Thus, searching for viable alternatives to prevent and treat infectious diseases is of great interest.

Currently, the therapeutic potential of plants has been recognized and explored. Studies have demonstrated the biological potential of isolated substances, extracts, and fractions obtained from different parts of the plants (leaves, bark, flowers, fruits, and roots) for different applications, such as antioxidants, anti-inflammatory, antitumor, against respiratory and digestive diseases, rheumatism, diabetes, and others [2].

Brazil is recognized for its extensive and exuberant biodiversity, still little explored [3]. Since 2009, Brazilian Ministry of Health has listed vegetable species in the National List of Medicinal Plants of Interest of the Unified Health System (Renisus), but only 71 plants were included by 2022 [4]. In addition to their application as phytotherapeutic agents and more accessible alternatives, plants stand out as a significant source of bioactive compounds, which can be used by industry to develop natural, semi-synthetic, or synthetic drugs [5].

Thus, it is essential to deepen the knowledge about medicinal plants, gather information about their properties and potential applications, as well as document the most recent studies to estimate the number and the profile of plants in the Brazilian biomes with biotechnological and pharmacological potential. Documenting this information may collaborate with the development of new antimicrobial products.

Thus, this work aimed to perform a scoping review of the literature about the antimicrobial activity of vegetable species found in Brazilian Biomes from 2016 to 2020 to document the current biotechnological potential of each biome and stimulate innovation. More than 90 Brazilian plant species were studied against 78 bacteria and 40 fungi strains, reuniting data may support future research in human health, especially those destined to study, develop, and produce new drugs with higher efficiency against resistant pathogens.

## 2. Results and Discussion

### 2.1. Brazilian Biomes and Plants with Antimicrobial Activity

**Figure 1:**
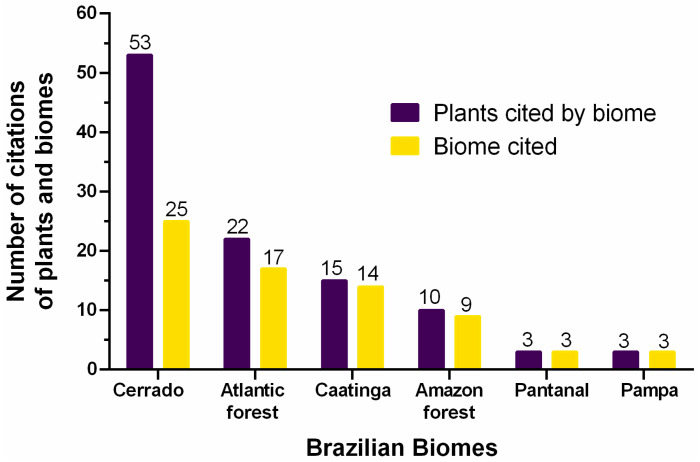
Number of plants cited per biome and articles citing each biome.

Brazil has six specific biomes: Atlantic forest, Amazon, Cerrado (or Savanna), Caatinga, and Pampa [6]. In this work, Cerrado was the biome with the highest number of collected plants and the most mentioned in the studies published from 2016 to 2020, followed by Atlantic Forest and Caatinga (Figure 1). Although the Amazon Forest is recognized as the largest biome in Brazil in area and biodiversity, the number of articles that study plants with antimicrobial activity from this biome was relatively small. It may suggest a lack of studies about the medicinal plants in that region, principally aiming to investigate their antimicrobial potential, as well as it may also be due to the difficult accessibility of the Amazonian biodiversity for scientific purposes.

Each biome has climatic and environmental peculiarities that influence biological variability. Cerrado, a savannah-like ecosystem, is the second largest biome in South America and occupies approximately 23.3% of the Brazilian territory, second only to Amazon, which covers 49.5% of the country [7]. It is formed by an environmental matrix, which composes the Brazilian central tablelands, with the most diverse flora in the planet, counting on almost 13 thousand vegetable species identified [8]. Cerrado physiognomies vary from grasslands to woodland, including the typical savanna (Cerrado *sensu stricto*), with different vegetation types [9]. This variability can influence the diversity of bioactive molecules from plants in different environmental, soil and water availability conditions [10].

According to Forzza [11], the Brazilian Cerrado was classified as a biodiversity hotspot for having over 12,669 catalogued plant species, from which 4215 are endemic. Besides, the high level of devastation towards Cerrado made this biome appear as one of the 36 richest and most threatened areas on the planet. Although hotspots represent only 17% of the Earth’s surface, these areas shelter 77% of all endemic plant species that exist in the planet, besides nearly 43% of mammals, birds, reptiles, and amphibia, which are endemic in these 36 regions [12]. This data reinforces the necessity to prioritize conservation to avoid the extinction of the threatened endemic species only described in Cerrado.

The biodiversity found in Cerrado is also source of subsistence to the families of several local communities that are part of the historical and cultural heritage in Brazil, mainly because they hold traditional knowledge of its biodiversity [13]. An example is the sustainable economic exploitation of the species *Dipteryx alata*, which is popularly known as *baruzeiro*. The seeds of this native plant are used as food and for phytotherapeutic agents [14].

The removal of the vegetation cover and the consequent loss of biodiversity leads to economic losses for Brazil once this biome has the potential to generate immeasurable bioeconomic resources but is still little explored [15]. It also threatens the existence of medicinal plants, which may contain still unknown active principles and could be efficient against resistant microorganisms.

Amazon and Atlantic forests have humid tropical climates and predominant vegetation [16,17]. The Atlantic Forest is the second largest forest in South America, one of the most biodiverse and threatened biomes in the world [18]. It is also a biodiversity hotspot, with only 11.6% of the natural vegetation cover remaining and evidence of a process of “savannization” [17]. Threats mainly affect the Atlantic Rainforest *sensu stricto*, the most heterogeneous part of the biome, a result of agricultural expansion, urbanization, and anthropogenic climate change [19]. This has attracted attention of scientists and environmentalists to the need for accompanying information from Palaeoecological research to bioeconomic capacity with sustainability.

Caatinga is characterized by a semi-arid climate and its vegetation is adapted to high temperature with low precipitation [20]. The number of studies on Caatinga plants found in this research is encouraging due to two main factors: (i) erroneous judgment that the ecosystem is poor in biodiversity and endemism; and (ii) during the dry season, the leaves fall and only the trunks of trees and shrubs remain. Although underestimated, Caatinga has unique species and characteristics, with several phytogeographical areas, and a significant number of rare endemic taxa [20]. Moro et al. [21] reported that a major component of Caatinga biodiversity are non-woody plants, comprising more than 60% of species in some local communities.

Pantanal (lowlands) has dry winters and rainy summers climate, and the predominant vegetation is floodplain [16]. It is the smallest Brazilian biome in terms of territory [16], but it is one of the largest wetlands in the world [22]. Anthropogenic changes that have taken place mainly in Amazon and Cerrado has been also affected the Pantanal, as soil erosion and difficult in water, food, and ecosystem service security [22]. However, subsistence practices and traditional medicine are common. Pessoa et al. [23] carried out a ethnobotanical study with 233 riverine people in the Pantanal regions and reported 32 botanical families with medicinal properties, mainly Fabaceae, Rutaceae, Amaranthaceae, and Solanaceae. The smaller number of articles found in this work on plants with antimicrobial activity emphasizes the need to expand studies and value small biomes.

Pampa is too a little biome, with a total area of 17,649,600 ha [16]. It has humid subtropical with hot summers climate, and the predominant vegetation is grasslands with bush formations [16]. The Pampa’s biodiversity includes medicinal plants important to conservations of the ecosystems and traditional communities.

The review of phytogeographic domains at *Flora do Brasil* platform showed that, from the 98 plants evaluated, 78 (86.63%) are native species in Brazil, 9 are naturalized, and 9 are cultivated in several Brazilian biomes (Table 1). The last 20 species correspond to endemic species in Brazil, and only 6 out of these cannot be found in the Brazilian Cerrado, confirming the relevance of this biome to preserving the Brazilian biodiversity. This may be related to the geographic position of the Cerrado in the Center-West of Brazil, with transition to virtually all other biomes.

The reported plants are distributed in 36 families, mainly Myrtaceae (13 citations), Fabaceae (12 citations), Lamiaceae (6 citations) and Lauraceae (6 citations). Myrtaceae family is one the main commercial fruit trees families, with 121 genus which fruits are a source of nutrients, antioxidants and compounds beneficial to health [24]. Fabaceae family includes legumes, one the largest group of flowering plant with species with medicinal properties [25].

**Figure 2:**
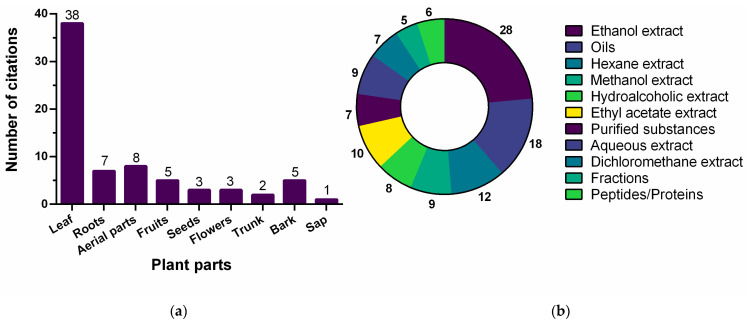
Total number of citations of the evaluated part of the plant (**a**) and the type of extract (**b**).

Medicinal plants have been used in different ways and with variable parts of the plants. In the present research, nine parts of the plants were usually cited: leaves, aerial parts, roots, fruits, seeds, flowers, stem, sap, and bark (Figure 2a). Seven different extracts were registered: ethanolic, hexanic, methanolic, hydro-alcoholic, ethyl acetate, dichloromethane and aqueous (Figure 2b). Besides, oils, isolated substances, fractions, and proteins/peptides were also detected (Figure 2b). Some articles reported the use of more than one part of the plant (9 parts resulting in a total of 72 citations) and more than one form of preparation (11 forms resulting in a total of 119 citations). The leaves and ethanolic extract were the most reported in the analyzed articles.

The significant use of leaves in the search for bioactive compounds with antimicrobial activity may be related to considerable concentration of substances by different extraction methods [26]. Besides, the leaves are abundant, its collection is not an invasive process that could damage or interfere with the plant life, and they are the most used part of plants in popular medicine to produce teas for therapeutic purposes. These findings corroborate with Liporacci and Simão [27], who performed an ethnobotanical study about medicinal plants in Ituiutaba City (Brazil) and observed that the leaves were the most used part (86.6%), followed by the roots (8.2%), flowers (3.4%), fruits (1%), seeds, and stem bark (0.8%).

The ethanolic extract was the most tested in the studies found. It is a solvent with very effective polarity, miscibility and viscosity characteristics for the extraction of phytochemicals [28]. It has been described for extracting alkaloids, saponins, carbohydrates, tanins, flavonoids, and phenolic compounds. Scientific literature reports that these substances have several medicinal properties, including anti-inflammatory, antiviral, antiparasitic, antifungal, and antibacterial potential [29]. The antimicrobial activity of ethanolic extracts has been reported to be greater than that of aqueous extracts due high content of polyphenols, as ethanol damage the cell wall more efficiently and improves extraction efficiency [30]. In addition, ethanol has a relatively low cost, low toxicity, and permission for use in the pharmaceutical and food industry by regulatory agencies in several countries when compared to other organic solvents, such as acetone and methanol [28].

### 2.2. Antifungal Activity

Fungal are eukaryotic microorganisms with an immense diversity of species, ubiquitous and associated with approximately 1.5 million deaths in humans annually [31]. Due to the increased resistance of fungal to clinically used drugs, the search for new antifungal molecules is necessary. Table 2 describes the antifungal activities of the main extracts, fractions, or isolated substances against 40 species of yeasts or filamentous fungi, sorted by the Minimum Inhibitory Concentration (MIC).

Most of the tested fungi are human pathogens. It is possible to identify that the yeasts *Candida albicans* and *Candida tropicalis* were the most tested strains, accounting for 47.76% (32 articles) and 20.89% (14 articles) of the citations, respectively. The genus *Candida* corresponds to opportunistic infectious agents, i.e., they cause infections in immunocompromised individuals. Candidemia is associated not only with high mortality rates (30 to 40%) but also with high hospital costs due to the long period of hospitalization [32].

The incidence of *Candida* infections has currently increased worldwide, and despite the availability of efficient antifungal agents, like the azole-antifungals and echinocandins, the mortality rates are still high. It may be related to the onset of resistant strains, which represent a major public health problem and a real threat to the infected patients [31,33]. An example is *Candida auris*, an emergent multidrug-resistant specie, widespread worldwide, and 90% of *C. auris* isolates are resistant to fluconazole in the USA [31]. Although *C. auris* has encouraged numerous studies for control methods, no article on medicinal plants tested against the strain was found in this search.

The genus *Fusarium* was also widely used as a model of filamentous fungus for the evaluation of antifungal activity, mainly for antimicrobial peptides. However, none of the products tested had a defined MIC, suggesting some difficulty in developing new agents against the genus. The genus *Fusarium* include both plant pathogens with economic impact and emergent opportunistic human pathogens associated to superficial and systemic infections [34]. In addition, the genus shows intrinsic resistance to several drugs and development of acquired resistance. New drugs effective against *Fusarium* are mainly synthetic or semi-synthetic substances, such as celecoxib derivates, orotomides, arylamidine derivates, and Hos2 histone deacetylase inhibitor, in clinical or preclinical development phase [34].

About the MIC values, we adopted interpretation parameter based on literature. The MIC values for pure or isolated substances were interpreted according to the proposed by Kuete [35], as follows: values <10 μg/mL indicated significant activity, values between 10 μg/mL and 100 μg/mL indicated moderate activity, and values >100 μg/mL were considered as low or insignificant activity. Extracts and fractions were interpreted according to Holetz et al. [36], as follows: values <100 μg/mL indicated significant activity, values between 100 μg/mL and 500 μg/mL were considered as moderate activity, 500 μg/mL to 1000 μg/mL indicated low activity, and values >1000 μg/mL were considered insignificant, irrelevant, or inactive.

Thus, the results represented in Table 2 show that 11 extracts, fractions and oils from plants had significant antifungal activity as they presented MIC values between 2.5 µg/mL and 62.5 µg/mL. About isolated substances, pure catechin obtained from *Banisteriopsis argyrophylla* (A. Juss) B. Gates (Malpighiaceae) leaves showed a significant effect against *Candida glabrata* (MIC 2.83 μg/mL), while 2 compounds showed moderate activity: valoneic acid from *Campomanesia adamantium* (Cambess.) O. Berg. (Myrtaceae) leaves [37] with MIC 15.62 µg/mL against *Trichophyton rubrum;* and lectin from the saline extract of *Portulaca elatior* Mart. ex Rohrb. (Portulacaceae) roots, with MIC 16.0 µg/mL against *Candida parapsilosis* [38]. Interestingly, in addition to activity for *T. rubrum,* valoneic acid was active on species of *Candida* e *Cryptococcus*, but any significant antibacterial activity was observed (MIC > 1000 µg/mL) [37].

Some of the vegetable species that stood out for their significant antifungal activity are *Eucalyptus globulus* Labill. (Myrtaceae), *Banisteriopsis argyrophylla* (A. Juss.) B. Gates (Malpighiaceae), *Caryocar coriaceum* Wittm. (Caryocaraceae), *Campomanesia adamantium* (Cambess.) O. Berg. (Myrtaceae), *Dypsis decaryi* (Jum.) Beentje & J. Dransf. (Arecaceae), *Portulaca elatior* Mart. ex Rohrb. (Portulacaceae), *Senna rugosa* (G. Don) H.S. Irwin & Barneby (Fabaceae), *Copaifera langsdorffii* Desf. (Fabaceae), and *Casearia sylvestris* Sw. (Salicaceae). These species showed significant to moderate activity against human pathogenic fungi under the experimental conditions evaluated by the authors.

**Table 2:** Minimum inhibitory concentration (MIC) and the number of citations for antifungal activity.

Strains	Citations	MIC (µg/mL)	Extract, Fraction, Bioactive Substance, and Plant
*Candida albicans*	32	2.5	Bioactive fractions F and J obtained from *Eucalyptus globulus* Labill. (Myrtaceae) stump wood methanolic extract [39].
*Candida tropicalis*	14	2.5	Bioactive fractions A and F obtained from *Eucalyptus globulus* Labill. (Myrtaceae) stump wood methanolic extract [39].
*Candida glabrata*	7	2.83	Fraction A1 (pure catechin) obtained from *Banisteriopsis argyrophylla* (A. Juss) B. Gates (Malpighiaceae) leaves [40].
*Microsporum canis*	2	4.1	Crude ethanolic extract obtained from the bark and fruit pulp of *Caryocar coriaceum* Wittm. (Caryocaraceae) [41].
*Candida krusei*	10	7.81	Aqueous fraction and aqueous Tannin of *Campomanesia adamantium* (Cambess) O. Berg. (Myrtaceae) leaves [37].
*Trichophyton mentagrophytes*	3	7.81	Volatile oil obtained from *Campomanesia adamantium* (Cambess.) O. Berg. (Myrtaceae) flowers [37].
*Sporotrichum schenckii*	3	10.35	Aqueous extract of *Dypsis decaryi* (Jum.) Beentje & J. Dransf. (Arecaceae) seed tegument [42].
*Cryptococcus neoformans*	1	15.62	Crude ethanolic extract and ethyl acetate fraction of *Campomanesia adamantium* (Cambess.) O. Berg. (Myrtaceae) leaves [37].
*Trichophyton rubrum*	2	15.62	Valoneic acid from *Campomanesia adamantium* (Cambess.) O. Berg. (Myrtaceae) leaves [37].
*Malassezia* spp.	1	39.1	Crude ethanolic extract of *Caryocar coriaceum* Wittm. (Caryocaraceae) fruit pulp and peel [41].
*Candida parapsilosis*	6	16.0	Lectin extracted from a saline extract of *Portulaca elatior* Mart. ex Rohrb. (Portulacaceae) roots [37,38].
*Candida famata*	2	62.5	Ethanolic extract of *Senna rugosa* (G. Don) H.S. Irwin & Barneby (Fabaceae) leaves [43].
*Paracoccidioides lutzii*	1	62.5	Copaíba resin oil obtained from the stem of *Copaifera langsdorffii* Desf. (Fabaceae) [44].
*Paracoccidioides brasiliensis*	1	62.5	Copaíba resin oil obtained from the stem of *Copaifera langsdorffii* Desf. (Fabaceae) [44].
*Paracoccidioides americana*	1	62.5	Copaíba resin oil obtained from the stem of *Copaifera langsdorffii* Desf. (Fabaceae) [44].
*Paracoccidioides restrepiensis*	1	62.5	Copaíba resin oil obtained from the stem of *Copaifera langsdorffii* Desf. (Fabaceae) [44].
*Saccharomyces cerevisae*	1	62.5	Non-oxygenated and oxygenated sesquiterpenes fraction 1–6, obtained from the leaves of *Casearia sylvestris* Sw. (Salicaceae) [45].
*Candida guilliermondii*	1	500 μg/disc	Aqueous extract obtained from *Eugenia dysenterica* (Mart) DC. (Myrtaceae) leaves [46].
*Microsporum gypseum*	1	500	Seed hexanic extract (soxhlet method) and seed ethanolic extract (maceration) of *Artocarpus heterophyllus* Lam. (Moraceae) [47].
*Epidermophyton floccosum*	1	1000	Seed hexanic extract (soxhlet method) of *Artocarpus heterophyllus* Lam. (Moraceae) [47].
*Rhizopus* sp.	1	1000	Seed hexanic extract (soxhlet method) of *Artocarpus heterophyllus* Lam. (Moraceae) [47].
*Malassezia furfur*	1	1800	Essential oils of *Piper augustum* Rudge (Piperaceae) leaves [48].
*Candida dubliniensis*	1	2500	Stem bark hexane extract of *Guatteria blepharophylla* Mart. (Annonaceae) [49].
*Aspergillus flavus*	1	Indefinite MIC	Leaves ethanolic extract of de *Davilla kunthii* A. St. –Hil. (Dilleniaceae) caused 11.92% of growth inhibition at 250 µg/mL [50].
*Fusarium proliferatum*	1	Indefinite MIC	Leaves ethanolic extract of de *Davilla kunthii* A. St. –Hil. (Dilleniaceae) caused 10.04% of growth inhibition at 250 µg/mL [50].
*Colletotrichum gloeosporioides*	4	Indefinite MIC	Proteic fraction denominated IIFF7Ca peptide, obtained from *Capsicum annuum* L. (Solanaceae) immature fruit caused 73.94% of growth inhibition at 200 μg/mL [51].
*Fusarium solani*	4	Indefinite MIC	Proteic fraction F5 (a peptide like Defensin) extracted from *Capsicum chinense* Jacq. (Solanaceae) fruits caused 44% of growth inhibition at 100 μg/mL [52].
*Fusarium oxysporum*	3	Indefinite MIC	Proteic fraction (trypsin inhibitor peptide *CaTI*) obtained from the seeds of *Capsicum annuum* L. (Solanaceae) caused growth inhibition at 64 µg/mL [35].
*Colletotrichum lindemuthianum*	2	Indefinite MIC	Proteic fraction (trypsin inhibitor peptide *CaTI*) obtained from the seeds of *Capsicum annuum* L. (Solanaceae) inhibited approximately 21% of growth inhibition at 64 µg/mL [53].
*Colletotrichum abscissum*	1	Indefinite MIC	Endophytic fungi extracts belonging to the *Diaporthe* cf. *heveae* LGMF1631 strain cultivated with malt extract obtained from the leaves and petiole of *Vochysia divergens* Pohl (Vochysiaceae) and *Stryphnodendron adstringens* (Mart.) Coville (Fabaceae) had 72% of mycelial growth inhibition [54].
*Fusarium verticillioides*	1	Indefinite MIC	Endophytic fungi extracts belonging to the *Diaporthe* cf. *heveae* LGMF1631 strain cultivated with malt extract obtained from the leaves and petiole of *Vochysia divergens* Pohl (Vochysiaceae) and *Stryphnodendron adstringens* (Mart.) Coville (Fabaceae) had 50% of mycelial growth inhibition [54].
*Phyllosticta citricarpa*	1	Indefinite MIC	Endophytic fungi extracts belonging to the *Diaporthe* cf. *heveae* LGMF1631 strain cultivated with malt extract obtained from the leaves and petiole of *Vochysia divergens* Pohl (Vochysiaceae) and *Stryphnodendron adstringens* (Mart.) Coville (Fabaceae) had 88% of mycelial growth inhibition [54].
*Fusarium lateritium*	1	Indefinite MIC	Proteic fraction Fa5 (antimicrobial peptide) obtained from *Capsicum annuum* L. (Solanaceae) fruits caused approximately 47% of growth inhibition [55].
*Sclerotinia sclerotiorum*	1	Indefinite MIC	Ethanol extract from the barks of *Byrsonima crassifolia* (L.) Kunth (Malpighiaceae) had 37.5% of mycelial growth inhibition at 24 µg/mL [56].
*Aspergillus fumigatus*	2	Inactive	Seed hexanic and ethanolic extracts of *Artocarpus heterophyllus* Lam. (Moraceae) at 1000 µg/mL [47]; Leaves ethanolic extract of *Peumus boldus* Molina (Monimiaceae)*;* leaves hydro-alcoholic extracts of *Psidium guajava* L. (Myrtaceae) and *Vernonia polysphaera* Baker (Asteraceae) at 500 mg/mL; dry leaves methanolic extract of *Persea americana* Mill. (Lauraceae) and raw sap of *Jatropha multifida* L. (Euphorbiaceae) at 1000 µg/mL [57]; Methanolic extract of *Simaba ferruginea* A.St-Hil. (Simaroubaceae) rhizome [58].
*Aspergillus niger*	1	Inactive	Leaves ethanolic extract of *Senna rugosa* (G. Don) H.S. Irwin & Barneby (Fabaceae) at 1000 µg/mL [43]; Methanolic extract of *Simaba ferruginea* A.St-Hil. (Simaroubaceae) rhizome [58].
*Aspergillus parasiticus*	1	Inactive	Methanolic extract of *Simaba ferruginea* A.St-Hil. (Simaroubaceae) rhizome [58].
*Aspergillus terreus*	1	Inactive	Methanolic extract of *Simaba ferruginea* A.St-Hil. (Simaroubaceae) rhizome [58].
*Penicillium expansum*	1	Inactive	Leaves ethanolic extract of *Senna rugosa* (G. Don) H.S. Irwin & Barneby (Fabaceae) at 1000 µg/mL [43].
*Ceratocystis cacaofunesta*	1	Inactive	Leaves aqueous and ethanolic solutions of *Adiantum latifolium* Lam. (Pteridaceae) [59].

The bioactive fractions obtained from the stump wood methanolic extract of *Eucalyptus globulus* Labill. (Myrtaceae) (popularly known as Eucalyptus) by column chromatography showed the best antifungal activity among the 14 fractions obtained in the study, with MIC values of 2.5 µg/mL against both *Candida albicans* and *Candida tropicalis* [39]. The sample is a source of bioactive polyphenols, also present antioxidant activity and the fractions were most effective than the crude extract.

The fruit peel and pulp crude ethanolic extract from *Caryocar coriaceum* Wittm. (Caryocaraceae), popularly known as Pequi, presented the best activity against *Microsporum canis* (MIC of 4.1 µg/mL), a filamentous fungi that infect domestic animals and occasionally humans [41]. Pequi is a fruit with expressive importance for the Brazilian population, especially in the central-west and southeast regions of the country. Fruit pulp is commonly used as food. In addition, fruit pulp and seeds oil are widely used for medicinal purposes as anti-inflammatory agents, wound healing, and for treating respiratory diseases like bronchial disorders, asthma, and cough. Its use is also reported against rheumatic and muscle pain and gastric ulcers [60]. Thus, scientific evidence about the medicinal properties of Pequi empower a production chain that develops the region and brings benefits to human health.

Volatile oil obtained from *Campomanesia adamantium* (Cambess.) O. Berg. (Myrtaceae) flowers presented significant activity against *Trichophyton mentagrophytes* [37]. This plant is popularly used for its anti-inflammatory, antidiarrheal, and urinary antiseptic activities. According to Sá et al. [37] the major constituents of the volatile oils from flowers were sabinene (20.45%), limonene (19.33%), α-thujene (8.86%), and methyl salicylate (8.66%), which support the described activities.

The species *Inga laurina* (Sw.) Willd (Fabaceae) also stood out due to its antifungal potential. The authors reported that the ethanolic extract and the ethyl acetate fraction obtained from its leaves had MICs of 11.7 µg/mL against *Candida glabrata*, while the n-butane fraction had MICs of 11.7 µg/mL against *Candida albicans* and 46.8 µg/mL against *Candida tropicalis* [61].

### 2.3. Antibacterial Activity

The prevalence of antibiotic-resistant bacteria has increased worldwide and it implies less availability of the drug to treat infections [62]. To have an idea, if no new antibiotic is developed may not be an effective drug available by 2050 [62]. Several control strategies have been described, such as modification of the current antimicrobials, combination therapy, phage therapy, nanotechnology-based systems, designing antimicrobial peptides, and use of natural compounds (or designing based on natural molecules) [63].

For millennia, medicinal plants have been used in traditional medicine and for over a century they have been used as a source or model for drugs, mainly in Western countries. The antibacterial activity of extracts, fractions, or isolated substances found in our research are represented in Table 3 and was organized according to their MIC values. Seventy-eight (78) bacteria strains were identified. All tested bacteria are recognized as human pathogens, most opportunistics. There are also bacteria of veterinary and agricultural importance, as *Rhodococcus equi* and *Kocuria rhizophila*, respectively.

*Staphylococcus aureus* was the most studied strain (54 articles, 80.60%), followed by *Escherichia coli* (49 articles, 73.13%), *Pseudomonas aeruginosa* (33 articles, 49.25%), *Staphylococcus epidermidis* (12 articles, 17.91%), and *Streptococcus mutans* (10 articles, 14.92%). *Staphylococcus aureus* is a Gram-positive, catalase- and coagulase-positive, non-fastidious bacteria, which is naturally present in human microbiota and generally causes no harm to immunocompetent individuals. However, it produces several toxins and expresses virulence factors that contribute to its capacity to invade organs and harm tissues, causing superficial diseases (such as folliculitis), systemic disorders, and syndromes that can cause death. It can also develop antibiotic resistance and become dangerous to patients in a hospital setting [64].

A well-known example is the methicillin-resistant Staphylococcus aureus (MRSA). The strains is characterized by acquision a Cassette Chromosome mec (SCCmec) containing mecA gene, which confers resistance to all β-lactam antibiotics [30]. Recently, anti-MRSA activities of several medicinal plants has been described. Okwu et al. [30] reported 51 medicinal plants collect from Thailand, Malaysia, India, Nigeria, Indonesia, China, South Africa and Peru with activities on MRSA. Most plants were prepared by extraction with ethanol and methanol, resulting extracts rich in phytochemicals as polyphenols, flavonoids, tannins, alkaloids and triterpenoids [30]. In this review, 3 natural products were found to act against MRSA: (1) a novel thermostable lectin from *Dypsis Decaryi* (Jum.) Beentje & J. Dransf. (Arecaceae) seeds [42]; (2) hydroethanolic extract from *Pereskia aculeata* Mill. (Cactaceae) leaves [65]; and (3) and fraction from the methanolic extract from *Eucalyptus globulus* Labill. (Myrtaceae) stump wood [39]. Among them, the product from *Eucalyptus* was the most effective.

About the MIC values, we adopted interpretation parameter based on literature [35,36], according to described for antifungal activity at 2.2. item. Over 28% of the tested bacterial strains had statistically significant susceptibility. The plants *Peritassa campestris* (Cambess.) A.C. Sm. (Celastraceae), *Portulaca elatior* Mart. ex Rohrb. (Portulacaceae), *Myrciaria pilosa* Sobral & Couto (Myrtaceae), *Campomanesia adamantium* (Cambess.) O. Berg. (Myrtaceae), *Rosmarinus officinalis* L. (Lamiaceae), *Chromolaena squalida* (DC.) RM King & H. Rob (Asteraceae), *Passiflora alata* Curtis (Passifloraceae), *Cochlospermum regium* (Mart. Et. Schr.) Pilger (Bixaceae), *Nectandra megapotamica* (Spreng.) Mez (Lauraceae), *Simaba ferruginea* A.St.-Hil. (Simaroubaceae), *Eugenia klotzschiana* O. Berg (Myrtaceae), *Croton heliotropiifolius* Kunth (Euphorbiaceae) and *Eucalyptus globulus* Labill. (Myrtaceae)stand out for their significant antibacterial activity, with MIC values varied from 0.78 μg/mL to 80 μg/mL, both for pure substances (<10 μg/mL) and for extracts and fractions (<100 μg/mL).

The results represented in Table 3 show that 15 extracts, fractions and oils from plants had moderate antibacterial activity (MIC values between 100 µg/mL and 500 µg/mL) and 5 had low activity (MIC values between 500 µg/mL and 1000 µg/mL. The antimicrobial activity evaluated for the 31 bacterial strains listed in Table 3 was considered insignificant, indeterminate, or inactive, with MIC values > 1000 μg/mL. These bacteria were mainly from the genus *Salmonella*, in addition to several strains of clinical isolates.

**Table 3:** Minimum inhibitory concentration (MIC) and number of citations for antibacterial activity.

Strains	Citations	MIC (µg/mL)	Extract, Fraction, Bioactive Substance, and Plant
*Bacillus megaterium*	1	0.78	Maytenin and maytenol isolated from the dichloromethane extract of *Peritassa campestris* (Cambess.) A.C. Sm. (Celastraceae) roots [66].
*Pseudomonas aeruginosa*	33	4.06	Lectin extracted from a saline extract of *Portulaca elatior* Mart. ex Rohrb. (Portulacaceae) roots [38].
*Staphylococcus aureus*	54	5.0	Essential oils of *Myrciaria pilosa* Sobral & Couto (Myrtaceae) leaves [67].
*Staphylococcus aureus* 679 *	1	5.0	Essential oils of *Myrciaria pilosa* Sobral & Couto (Myrtaceae) leaves [67].
*Staphylococcus aureus* 683 **	1	5.0	Essential oils of *Myrciaria pilosa* Sobral & Couto (Myrtaceae) leaves [67].
*Staphylococcus* sp. 841 ***	1	7.80	Essential oils of *Chromolaena squalida* (DC.) RM King & H. Rob (Asteraceae) leaves [15].
*Enterococcus faecalis*	6	8.12	Lectin extracted from saline extract of *Portulaca elatior* Mart. ex Rohrb. (Portulacaceae) roots [38].
*Bacillus thuringiensis*	1	9.08	Crude extract from the roots of *Passiflora alata* Curtis (Passifloraceae) [68].
*Staphyloccocus epidermidis*	12	16	Ethanolic extract and fractions from dichloromethane of *Rosmarinus officinalis* L. (Lamiaceae) leaves [69].
*Streptococcus sobrinus*	4	20	Essential oils of *Nectandra megapotamica* (Spreng.) Mez (Lauraceae) leaves [70]
*Escherichia coli*	49	25	Methanolic extract of *Simaba ferruginea* A.St.-Hil. (Simaroubaceae) rhizome [58].
*Streptococcus pyogene*	2	28.98	Crude extract from the roots of *Passiflora alata* Curtis (Passifloraceae) [68].
*Listeria monocytogenes*	6	31.25	Volatile oile from the leaves of *Campomanesia adamantium* (Cambess.) O. Berg. (Myrtaceae) [37].
*Acinetobacter baumannii*	2	31.25	Isolated tannic acid-enriched fraction from the roots of *Cochlospermum regium* (Mart. Et. Schr.) Pilger (Bixaceae) [71].
*Listeria innocua*	1	31.25	Dichloromethane fraction of *Campomanesia adamantium* (Cambess.) O. Berg. (Myrtaceae) leaves [37].
*Bacteroides fragilis*	1	31.25	Essential oils of *Nectandra megapotamica* (Spreng.) Mez (Lauraceae) leaves [70].
*Bacillus cereus*	6	32	Ethanolic extract and fractions from dichloromethane of *Rosmarinus officinalis* L. (Lamiaceae) leaves [69].
*Streptococcus mutans*	10	50	Essential oils of *Eugenia klotzschiana* O.Berg (Myrtaceae) leaves in natura, dry leaves, and flowers [72]; Essential oils of *Nectandra megapotamica* (Spreng.) Mez (Lauraceae) leaves [70].
*Prevotella nigrescens*	1	50	Essential oils of *Eugenia klotzschiana* O.Berg (Myrtaceae) leaves in natura, dry leaves, and flowers [72]; Essential oils of *Nectandra megapotamica* (Spreng.) Mez (Lauraceae) leaves [70].
*Enterobacter cloacae*	2	62.5	Hexane fraction of *Campomanesia adamantium* (Cambess.) O. Berg. (Myrtaceae) leaves [37].
*Bacillus subtilis*	2	62.5	Essential oils from *Croton heliotropiifolius* Kunth (Euphorbiaceae) aerial parts (leaves and stems) [73].
*Klebsiella pneumoniae*	9	80	Bioactive fraction I of the methanolic extract from the stump wood of *Eucalyptus globulus* Labill. (Myrtaceae) [39].
*Porphyromonas gingivalis*	1	100	Hydroalcoholic extract of *Copaifera trapezifolia* Hayne (Fabaceae) leaves [74].
*Peptostreptococcus micros* (Clinical isolate)	1	100	Hydroalcoholic extract of *Copaifera trapezifolia* Hayne (Fabaceae) leaves [74].
*Micrococcus luteus*	3	125	Hexane fraction of *Campomanesia adamantium* (Cambess.) O. Berg. (Myrtaceae) leaves [37].
*Micrococcus roseus*	1	125	Hexane fraction of *Campomanesia adamantium* (Cambess.) O. Berg. (Myrtaceae) leaves [37].
*Streptococcus agalactiae*	2	125	Essential oil of *Mentha piperita* L. (Lamiaceae) leaves [75].
Methicillin-resistant *Staphylococcus aureus*	3	156	Bioactive fraction I obtained from the methanolic extract of *Eucalyptus globulus* Labill. (Myrtaceae) stump wood [39].
*Staphylococcus haemolyticus*	1	170	Ethanolic extract of *Schinopsis brasiliensis* Engl. (Anacardiaceae) leaves [76].
*Streptococcus salivarius*	2	200	Essential oils of *Eugenia klotzschiana* O.Berg (Myrtaceae) leaves *in natura*, dry leaves, and flowers [72].
*Streptococcus salivarius* (Clinical isolate)	1	200	Hydroalcoholic extract of *Copaifera trapezifolia* Hayne (Fabaceae) leaves [74].
*Shigella flexneri*	3	200	Methanolic extract of *Simaba ferruginea* A.St.-Hil. (Simaroubaceae) rhizome [58].
*Enterobacter aerogenes*	1	250	Dichloromethane fraction of *Campomanesia adamantium* (Cambess.) O. Berg. (Myrtaceae) leaves [37].
*Staphylococcus* sp. 873 ****	1	250	Essential oils from the leaves of *Chromolaena squalida* (DC.) RM King & H. Rob (Asteraceae); *Campomanesia sessiliflora* (O. Berg) Mattos (Myrtaceae); *Ocotea minarum* (Nees & Mart.) Mez (Lauraceae), and *Endlicheria paniculata* (Spreng.) JF Macbr. (Lauraceae) [15].
*Salmonella* sp. ATCC 6017	2	200–400	Essential oil *GB2a* of *Ocimum basilicum* L. aerial parts (Lamiaceae), known as Green Brazil [77].
*Salmonella enterica*	3	390	Crude ethanolic extract from the *Schinopsis brasiliensis* Engl. (Anacardiaceae) barks [78].
*Streptococcus sanguinis*	3	400	Essential oils of *Eugenia klotzschiana* O.Berg (Myrtaceae) leaves *in natura*, dry leaves, and flowers [72].
*Streptococcus sanguinis* (Clinical isolate)	1	200	Hydroalcoholic extract of *Copaifera trapezifolia* Hayne (Fabaceae) leaves [74].
*Actinomyces naeslundii* (Clinical isolate)	1	400	Hydroalcoholic extract of *Copaifera trapezifolia* Hayne (Fabaceae) leaves [74].
*Fusobacterium nucleatum*	1	400	Hydroalcoholic extract of *Copaifera trapezifolia* Hayne (Fabaceae) leaves [74].
*Streptococcus mitis*	1	>400	Hydroalcoholic extract of *Copaifera trapezifolia* Hayne (Fabaceae) leaves [74].
*Lactobacillus casei*	1	>400	Hydroalcoholic extract of *Copaifera trapezifolia* Hayne (Fabaceae) leaves [74].
*Prevotella intermedia*	1	>400	Hydroalcoholic extract of *Copaifera trapezifolia* Hayne (Fabaceae) leaves [74].
*Actinomyces viscosus* (Clinical isolate)	1	>400	Hydroalcoholic extract of *Copaifera trapezifolia* Hayne (Fabaceae) leaves [74].
*Prevotella buccae* (Clinical isolate)	1	>400	Hydroalcoholic extract of *Copaifera trapezifolia* Hayne (Fabaceae) leaves [74].
*Porphyromonas gingivalis* (Clinical isolate)	1	>400	Hydroalcoholic extract of *Copaifera trapezifolia* Hayne (Fabaceae) leaves [74].
*Actinomyces naeslundii*	1	>400	Hydroalcoholic extract of *Copaifera trapezifolia* Hayne (Fabaceae) leaves [74].
*Listeria grayi*	1	450	Essential oils of *Hedychium coronarium* J.Koenig (Zingiberaceae) leaves [48].
*Salmonella typhi* 905 *****	1	500	Essential oils of *Campomanesia sessiliflora* (O. Berg) Mattos (Myrtaceae) leaves [15].
*Kocuria rhizophila*	1	>500	Galangin-3-methyl ether isolated from the aerial parts of *Lychnophora markgravii* G.M. Barroso (Asteraceae) [79].
*Providencia stuartii*	1	750	DdeL—A new thermostable lecitin extracted from the seeds of *Dypsis decaryi* (Jum.) Beentje & J. Dransf. (Arecaceae) [42].
*Enterococcus hirae*	1	900	Hydro-ethanolic extract of *Myrcia fallax* (Rich.) DC. (Myrtaceae) leaves [80].
*Klebsiella oxytoca*	2	900	Essential oils of *Hedychium coronarium* J.Koenig (Zingiberaceae) leaves [48].
*Proteus vulgaris*	1	900	Essential oils of *Hedychium coronarium* J.Koenig (Zingiberaceae) leaves [48].
*Rhodococcus equi*	1	1000	Hydro-ethanolic extract of *Myrcia guianensis* (Aubl.) DC. (Myrtaceae) leaves [80].
*Enterococcus faecalis* clinical isolate	4	1500	DdeL—A new thermostable lecitin extracted from the seeds of *Dypsis decaryi* (Jum.) Beentje & J. Dransf. (Arecaceae) [42].
Extended spectrum beta-lactamase-producing (ESBL) Enterobacteriaceae (Clinical isolate)	1	1500	DdeL—A new thermostable lecitin extracted from the seeds of *Dypsis decaryi* (Jum.) Beentje & J. Dransf. (Arecaceae) [42].
*Morganella morganii*	1	20,000	Hydro-ethanolic extract of *Pereskia aculeata* Mill. (Cactaceae) leaves [65].
*Proteus mirabilis*	2	>20,000	Hydro-ethanolic extract of *Pereskia aculeata* Mill. (Cactaceae) leaves [65].
*Salmonella typhimurium*	5	25,000	Ethyl acetate extract of *Ocotea silvestris* Vattimo-Gil (Lauraceae) leaves [81].
*Salmonella enteritidis*	3	25,000	Alcoholic and ethyl acetate extracts of *Ocotea silvestris* Vattimo-Gil (Lauraceae) leaves [81].
*Salmonella heidelberg*	1	25,000	Alcoholic and ethyl acetate extracts of *Ocotea silvestris* Vattimo-Gil (Lauraceae) leaves [81].
*Salmonella infantis*	1	25,000	Alcoholic extract of *Ocotea silvestris* Vattimo-Gil (Lauraceae) leaves [81].
*Salmonella ohio*	1	25,000	Alcoholic extract of *Ocotea silvestris* Vattimo-Gil (Lauraceae) leaves [81].
*Salmonella naintpaul*	1	25,000	Alcoholic extract of *Ocotea silvestris* Vattimo-Gil (Lauraceae) leaves [81].
*Salmonella agona*	1	50,000	Aqueous, alcoholic, and ethyl acetate extracts of *Ocotea silvestris* Vattimo-Gil (Lauraceae) leaves; Aqueous and ethyl acetate extracts of *Ocotea diospyrifolia* (Meisn.) Mez (Lauraceae) leaves [81].
*Salmonella mbandaka*	1	50,000	Aqueous, alcoholic, and ethyl acetate extracts of *Ocotea silvestris* Vattimo-Gil (Lauraceae) leaves; Aqueous and ethyl acetate extracts of *Ocotea diospyrifolia* (Meisn.) Mez (Lauraceae) leaves [81].
*Salmonella gallinarum*	1	50,000	Alcoholic and ethyl acetate extracts of *Ocotea silvestris* Vattimo-Gil (Lauraceae) leaves; ethyl acetate extract of *Ocotea diospyrifolia* (Meisn.) Mez (Lauraceae) leaves [81].
*Salmonella give*	1	50,000	Alcoholic and ethyl acetate extracts of *Ocotea silvestris* Vattimo-Gil (Lauraceae) leaves; ethyl acetate extract of *Ocotea diospyrifolia* (Meisn.) Mez (Lauraceae) leaves [81].
*Serratia marcescens*	1	62,500	Crude ethanolic extract from the *Schinopsis brasiliensis* Engl. (Anacardiaceae) barks [78].
*Staphylococcus sanguinis*	2	Indeterminate MIC	Ethanolic extract of *Davilla kunthii* A. St. –Hil. (Dilleniaceae) leaves at 9.375 µg/mL caused 21,198% of growth inhibition [50].
*Stenotrophomonas maltophilia*	1	Indeterminate MIC	Endophytic fungi extracts belonging to the *Diaporthe* cf. *heveae* LGMF1631strain cultivated with malt extract obtained from the leaves and petiole of *Vochysia divergens* Pohl (Vochysiaceae) and *Stryphnodendron adstringens* (Mart.) Coville (Fabaceae) showed an inhibition halo of 16 mm [54].
*Aeromonas caviae*	1	Inactive	Crude extract from the leaves and roots of *Passiflora alata* Curtis (Passifloraceae); *Passiflora foetida* L. (Passifloraceae); *Passiflora pohlii* Mast. (Passifloraceae), and *Passiflora suberosa* L. (Passifloraceae) at 1000 µg/mL [68].
*Aeromonas hydrophila*	1	Inactive	Crude extract from the leaves and roots of *Passiflora alata* Curtis (Passifloraceae); *Passiflora foetida* L. (Passifloraceae); *Passiflora pohlii* Mast. (Passifloraceae), and *Passiflora suberosa* L. (Passifloraceae) at 1000 µg/mL [68].
*Citrobacter freundii*	1	Inactive	*Passiflora alata* Curtis (Passifloraceae); *Passiflora foetida* L. (Passifloraceae); *Passiflora pohlii* Mast. (Passifloraceae), and *Passiflora suberosa* L. (Passifloraceae) at 1000 µg/mL [68].
*Shigella sonnei*	1	Inactive	Crude extract from the leaves and roots of *Passiflora alata* Curtis (Passifloraceae); *Passiflora foetida* L. (Passifloraceae); *Passiflora pohlii* Mast. (Passifloraceae), and *Passiflora suberosa* L. (Passifloraceae) at 1000 µg/mL [68].
*Staphylococcus saprophyticus*	1	Inactive	Crude extract from the leaves and roots of *Passiflora alata* Curtis (Passifloraceae); *Passiflora foetida* L. (Passifloraceae); *Passiflora pohlii* Mast. (Passifloraceae), and *Passiflora suberosa* L. (Passifloraceae) at 1000 µg/mL [68].
*Helicobacter pylori*	1	Inactive	Hydroethanolic extract of *Piper umbellatum* L.(Piperaceae) leaves [82].

* Clinical isolate penicillin resistant. ** Clinical isolate penicillin, cephalothin, cefoxitin, ciprofloxacin and clindamycin resistant. *** Coagulase-positive *Staphylococcus* sp. Veterinary clinical strain (resistant to ampicillin, doxycycline, clindamycin, cefoxitin). **** Veterinary clinical strain (resistant to ampicillin, doxycycline, clindamycin, penicillin, oxacillin, norfloxacin, cefoxitin, azithromycin). ***** Veterinary clinical strain (resistant to clindamycin, penicillin, oxacillin).

The compounds maytenin and maytenol isolated from the root dichloromethane extract of *Peritassa campestris* (Cambess.) A.C. Sm. (Celastraceae), popularly known as Bacupari, presented the lowest MIC found in our research (0.78 μg/mL, Table 3) against *Bacillus megaterium*. This strain corresponds to a Gram-positive, preferentially aerobic, and non-pathogenic bacteria found in the soil but can colonize and benefit some vegetable tissues (endophyte), like protecting wheat from developing fungi disease caused by *Septoria tritici*, for example [83]. The result shows the potential for application in pest control in agriculture.

*Portulaca elatior* Mart. ex Rohrb. (Portulacaceae) is a specie native from Caatinga, Cerrado and Atlantic Forest biomes. A trehalose-binding lectin (PeRoL) was isolated from *P. elatior* root extract [38]. It is an acidic and thermostable protein, the first isolated from that plant, and presented an bacteriostatic activity against *Pseudomonas aeruginosa*, *Enterococcus faecalis,* and *Staphylococcus aureus* (MIC of 4.06, 8.12, and 32.5 μg/mL, respectively). In addition, the compound was not cytotoxic to human peripheral blood mononuclear cells and human erythrocytes.

*Myrciaria pilosa* Sobral & Couto (Myrtaceae) is a plant native from Caatinga and Atlantic Forest biomes. The essential oil extracted from its leaves rich in sesquiterpenes guaiol and β-caryophyllene was described for the first time for antibacterial and antiviral activities [67]. The oil inhibited the growth of standard strain and clinical isolates of Staphylococcus aureus at 5 μg/mL.

Other plant species deserve to be highlighted, despite not having presented the lowest MIC compared to the plants mentioned in Table 3. All the following plants occur naturally in the Cerrado biome. Essential oil from *Campomanesia sessiliflora* (O. Berg) Mattos (Myrtaceae) and *Myrsine guianensis* (Aubl.) Kuntze (Primulaceae) presented MIC of 31.25 μg/mL against *Staphylococcus aureus* resistant to ampicillin, doxycycline, clindamycin, and cefoxitin, isolated from cows with mastitis [15]. The result shows the potential for application in pest control in the livestock sector with a strong economic impact.

Additionally, the crude extract from the roots of *Passiflora foetida* L. presented MIC of 36.89 μg/mL against *Streptococcus pyogenes* and 37.51 μg/mL against *Bacillus thuringiensis* [68]. *Cochlospermum regium* (Mart. Et. Schr.) Pilger (Bixaceae) showed antibacterial and antifungal activities, associated with tannin and gallic acid content. Tannic acid-enriched fraction from *C. regium* roots present MIC of 31.25 µg/mL to *Staphylococcus epidermidis* [71]. These data support the folk use and possibles models to development of antimicrobials. Essential oil from *Ocimum canum* Sims. (Lamiaceae) leaves presented MIC of 100 µg/mL against *Pseudomonas aeruginosa* [84]. The activity was attributed to compound thymol, abundant in that oil and reported in scientific literature as antimicrobial.

### 2.4. Bioactive Molecules and Mechanisms of Action

The antibacterial and antifungal activity observed for some plants presented in our research may be related to the presence of active principles produced by the secondary metabolism of these plants. Secondary metabolites are a group of substances produced by secondary metabolism of the plant, which are not essential for growth and life, but protect against animals and microorganisms. They are generally classified in three groups: phenolic compounds, terpenes, and nitrogen compounds [85]. Phenolic compounds are characterized by one or more hydroxylated aromatic ring and one of the main classes is the flavonoid; terpenoids are polymeric isoprene; and alkaloids are non-protein nitrogen-containing molecules, although it is synthesized from amino acids [86].

The association of these compounds with beneficial health effects is not new. The alkaloid quinine was widely used to treat parasitic and bacterial disease and cinnamaldehyde was very used to treat diverse infections in Chinese or Indian medicine [86]. The compounds most cited by the articles analyzed in the present study, which could be responsible for the antimicrobial activities reported, were: (i) flavonoids (tannins, catechins, quercetin, rutin, gallic acid, and caffeic acid), (ii) triterpenoids, monoterpenoid (thymol) and sesquiterpenes (spathulenol, α-copaene, and β-caryophyllene), which are part of the terpenes group; and (iii) alkaloids, representing the nitrogen compounds.

The antimicrobial activity displayed by these compounds may happen through several mechanisms of action. In general, the plant compounds exert antibacterial activity by: (i) DNA and protein synthesis inhibitor, including inhibition of DNA gyrase enzyme; (ii) efflux pump inbibitor; (iii) cell membrane disturbance or damage; (iv) outer membrane damage; (v) inhibition of the cell division protein, as FtsZ; (vi) inhibition of enzyme required for biosynthesis of peptidoglycan; (vii) inhibition of enzymes required for biosynthesis of type II fatty acid [86].

The antifungal mechanisms of action by secondary metabolites include inhibition of cell wall formation; cell membrane disruption; dysfunction of the fungal mitochondria as inhibition of electron transport or ATPase enzyme; inhibition of cell division; inhibition of RNA/DNA or protein synthesis; and inhibition of efflux pumps [87]. In general, terpenes compounds affect fungal cell membrane and mitochondria, phenolics compounds act on cell wall, cell membrane and mitochondria, while alkaloids affect mainly cell wall and membrane [87].

The antifungal activity of compounds like gallic acid and tannic acid extracted from the ethyl acetate fraction obtained from the roots of *Cochlospermum regium* (mart. Et. Schr.) Pilger (popularly known as algodãozinho do campo) for example, may be related to the linkage of these compounds to membrane ergosterol, leading to membrane disruption and intracellular content leakage [71]. The antibacterial activity for the same sample may be related to the inhibition of catalase, leading to the accumulation of reactive oxygen species (ROS) and, consequently, cell damage and death [71].

Lima et al. [88] claim that phenolic compounds-rich extracts can display a broad-spectrum antibacterial activity, even against pathogenic microorganisms. This activity may happen through membrane permeability alterations as well as polarization and efflux interruption, generating severe cell damage and leading to cell death. Sarpietro et al. [89] reported that the antifungal potential of sesquiterpene compounds, like β-caryophyllene, may be displayed by the release of membrane lipophilic substances when this compound is absorbed by the fungi. Kuete [35] reported that the antibacterial and antifungal activities of quinones is through the irreversible complexation of microbial nucleophilic amino acid, leading to function loss and cell death.

Flavonoids has been described as inhibitory to efflux pump on Gram-negative bacteria *Escherichia coli* [86]. This sheds light on control of main mechanisms of resistance to antimicrobial drugs by bacteria. The efflux pump is, for example, the mechanism which carbapenems drugs are excluded from periplasmic space, while over expressions of the naturally existing pump create mutations in the DNA gyrase and topoisomerase IV enzymes, important targets to antimicrobials [63]. In addition, d-alanine:d-alanine ligase (Ddl) was reported as a new target for the flavonoids quercetin and apigenin, acting as competitive inhibitors with the substrate ATP [90]. This is important to control the mechanisms of resistance for tetracycline, which include energy-dependent efflux pumps (ABC efflux pumps) [63].

## 3. Materials and Methods

**Figure 3:**
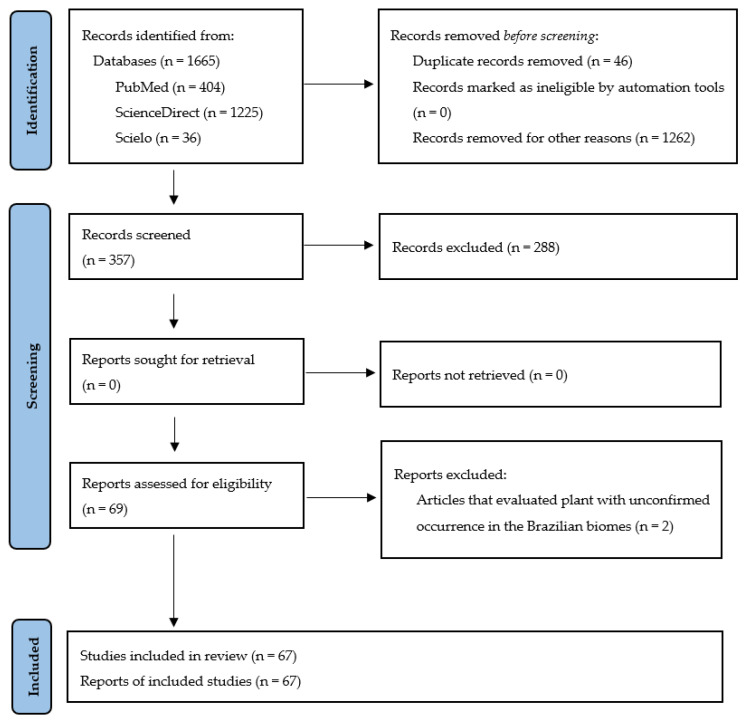
Flow diagram of study selection.

This study is a descriptive and quantitative scoping review of the literature about the antimicrobial activity of plants found in Brazilian biomes. This searches of databases, registers and selection of works were conducted in accordance with the PRISMA (Preferred Reporting Items for Systematic Reviews and Meta-Analyses) guidelines (Figure 3). The search was performed using PubMed, ScienceDirect and Scielo databases applying the following descriptors in English: vegetal extract, antimicrobial, antifungal, antibacterial, Cerrado, Amazon, Atlantic Forest, Caatinga, Pantanal, Pampa, and Brazil. In Portuguese, the descriptors used were *extrato vegetal*, *antimicrobiano*, *Cerrado*, *Amazônia, Mata Atlântica, Caatinga, Pantanal, Pampa* and *Brasil*. The descriptors were applied in random combinations. The publication period selected was 2016 to 2020.

The inclusion criteria were as follows: scientific articles of original experimental research, studies about plant extracts, fractions, or isolated substances, and studies comprising antibacterial and antifungal assays. The exclusion criteria were literature reviews, thesis, dissertations, repeated articles, and studies without antibacterial or antifungal assays.

At first, the titles and abstracts of the studies found were analyzed. After that, the full content of the articles was evaluated to gather information. The application of inclusion and exclusion criteria resulted in 357 publications. After reading the titles and abstracts, 69 articles were selected for thorough reading to verify if they were aligned with the objective of the study and, if so, extract the data of interest. After full reading, two articles were excluded once they evaluated three plant species with unconfirmed occurrence in the Brazilian biomes, despite the studies being performed in Brazil. Thus, the results reported in the present study were obtained from 67 articles, 2 in Portuguese and 65 in English.

The results of these analyzes were organized, registering the number of publications found in each database, the number of publications per year, the main species of plants as well as the main species of bacteria and fungi tested, plants preparation (isolation of substances, preparation of extracts and fractions), the parts of the plants used, bioactive compounds identified, and the Minimum Inhibitory Concentrations (MIC). The MICs were considered when obtained by in vitro methods of evaluating the inhibition of growth of bacteria and fungi, such as: microdilution method in 96-well microplates, macrodilution method, resazurin microtiter and disk diffusion method. Most MICs were determined using Clinical & Laboratory Standards Institute (CSLI) methodologies. The species names and phytogeographic domains were confirmed at *Flora do Brasil* platform [91]. Tables were organized with Excel^®^ and the graphs were constructed with the software GraphPad Prism 8 (GraphPad Softwares, EUA).

## 4. Conclusions

The present study showed several species belonging to Brazilian biodiversity with antimicrobial medicinal properties and promising results for the development of potent and efficient products towards resistant micro-organisms. The species *Eucalyptus globulus* Labill. (Myrtaceae), *Banisteriopsis argyrophylla* (A. Juss.) B. Gates (Malpighiaceae) *Caryocar coriaceum* Wittm. (Caryocaraceae), *Campomanesia adamantium* (Cambess.) O. Berg. (Myrtaceae), *Dypsis decaryi* (Jum.) Beentje & J. Dransf. (Arecaceae), *Portulaca elatior* Mart. ex Rohrb. (Portulacaceae), *Senna rugosa* (G. Don) H.S. Irwin & Barneby (Fabaceae), *Copaifera langsdorffii* Desf. (Fabaceae), *Casearia sylvestris* Sw. (Salicaceae) and *Inga laurina* (Sw.) Willd. (Fabaceae) stood out against fungi.

On the other hand, *Peritassa campestris* (Cambess.) A.C. Sm. (Celastraceae), *Portulaca elatior* Mart. ex Rohrb. (Portulacaceae), *Myrciaria pilosa* Sobral & Couto (Myrtaceae), *Campomanesia adamantium* (Cambess.) O. Berg. (Myrtaceae), *Rosmarinus officinalis* L. (Lamiaceae), *Chromolaena squalida* (DC.) RM King & H. Rob (Asteraceae), *Passiflora alata* Curtis (Passifloraceae), *Cochlospermum regium* (Mart. Et. Schr.) Pilger (Bixaceae), *Nectandra megapotamica* (Spreng.) Mez (Lauraceae), *Simaba ferruginea* A.St.-Hil. (Simaroubaceae), *Eugenia klotzschiana* O. Berg (Myrtaceae), *Croton heliotropiifolius* Kunth (Euphorbiaceae) and *Eucalyptus globulus* Labill. (Myrtaceae) stood out against pathogenic bacteria.

Most of those plants occur naturally in the Cerrado biome. It is noteworthy that the Brazilian Cerrado has considerable biotechnological potential, but environmental damages threat the maintenance of vegetable species with reported antimicrobial activity as well as other species with unknown antimicrobial potential. Thus, it is important to invest in public policies focused on protecting and preserving biome and research for bioprospecting more vegetable species with potential to generate bioeconomic wealth in Brazil. Substances with promising biological activity may represent an alternative to reduce economic losses generated by the high cost of hospital admissions due to infections caused by pathogenic microorganisms.

## Figures and Tables

**Table 1:** Origin and distribution with phytogeographic domains of the prospected vegetable species studied for antimicrobial applications.

Species and Family	Origin	Distribution—Phytogeographic Domains					
		Amazon	Caatinga	Cerrado	Atlantic Forest	Pampa	Pantanal
*Amburana cearensis* (Allemão) A.C.Sm. (Fabaceae)	Native		X	X	X		X
*Acrocomia aculeata* (Jacq.) Lodd. Ex Mart. (Arecaceae)	Native			X	X		
*Adiantum latifolium* Lam. (Pteridaceae)	Native	X			X		
*Annona coriacea* Mart. (Annonaceae)	Native	X	X	X			X
*Annona crassiflora* Mart. (Annonaceae)	Native	X		X			X
*Arrabidaea brachypoda* (DC.) bureau (Bignoniaceae)	Native	X	X	X	X		X
*Artocarpus heterophyllus* Lam. (Moraceae)	Naturalized	X	X		X		
*Aspidosperma subincanum* Mart. (Apocynaceae)	Native	X		X	X		
*Banisteriopsis argyrophylla* (A.Juss.) B.Gates (Malpighiaceae)	Native			X			
*Bauhinia rufa* (Bong.) Steud. (Fabaceae)	Native			X			
*Brosimum gaudichaudii* Trécul. (Moraceae)	Native	X	X	X	X		
*Byrsonima crassifolia* (L.) Kunth (Malpighiaceae)	Native	X	X	X	X		X
*Campomanesia adamantium* (Cambess.) O. Berg. (Myrtaceae)	Native			X	X		
*Campomanesia sessiliflora* (O. Berg) Mattos (Myrtaceae)	Native		X	X	X		
*Capsicum annuum* L. (Solanaceae)	Cultivated	X					
*Capsicum baccatum* L. (Solanaceae)	Native	X	X	X	X	X	X
*Capsicum chinense* Jacq. (Solanaceae)	Naturalized	X					
*Capsicum frutescens* L. (Solanaceae)	Naturalized	X			X		
*Caryocar brasiliense* Cambess. (Caryocaraceae)	Native	X	X	X	X		
*Casearia sylvestris* Sw. (Salicaceae)	Native	X	X	X	X	X	X
*Chromolaena squalida* (DC.) R.M.King & H.Rob. (Asteraceae)	Native	X	X	X	X		
*Cochlospermum regium* (Mart. Ex Schrank) Pilg. (Bixaceae)	Native	X	X	X			X
*Copaifera langsdorffii* Desf. (Fabaceae)	Native	X	X	X	X		
*Copaifera trapezifolia* Hayne (Fabaceae)	Native				X		
*Croton heliotropiifolius* Kunth (Euphorbiaceae)	Native		X	X	X		
*Davilla kunthii* A.St.-Hil. (Dilleniaceae)	Native	X		X	X		
*Dipteryx alata* Vogel (Fabaceae)	Native			X			
*Dypsis decaryi* (Jum.) Beentje & J.Dransf. (Arecaceae)	Cultivated	No phytogeographic domain—Confirmed occurrence in the Brazilian southeast					
*Endlicheria paniculata* (Spreng.) J.F.Macbr. (Lauraceae)	Native	X	X	X	X	X	X
*Erythroxylum daphnite* daphnites Mart. (Erythroxylaceae)	Native	X	X	X	X		
*Eucalyptus citriodora* Hook. (Myrtaceae)	Cultivated	Occurrence: North, Northeast, Central-West, Southeast, and South					
*Eucalyptus globulus* Labill. (Myrtaceae)	Cultivated	Occurrence: North, Northeast, Central-West, Southeast, and South					
*Eugenia klotzschiana* O.Berg (Myrtaceae)	Native			X	X		
*Genipa americana* L. (Rubiaceae)	Native	X	X	X	X		X
*Guatteria blepharophylla* Mart. (Annonaceae)	Native	X					
*Hedychium coronarium* J.Koenig (Zingiberaceae)	Naturalized	X	X	X	X	X	X
*Inga laurina* (Sw.) Willd. (Fabaceae)	Native	X	X	X	X		
*Iryanthera polyneura* Ducke (Myristicaceae)	Native	X					
*Jatropha multifida* L. (Euphorbiaceae)	Cultivated	Occurrence: North, Northeast, Southeast, and South					
*Matayba guianensis* Aubl. (Sapindaceae)	Native	X		X	X		X
*Mauritia flexuosa* L.f. (Arecaceae)	Native	X	X	X			
*Melissa officinalis* L. (Lamiaceae)	Cultivated	Occurrence: North, Northeast, Central-West, Southeast, and South					
*Mentha piperita* L. (Lamiaceae)	Cultivated	No information found					
*Myrcia bella* Cambess. (Myrtaceae)	Native			X			
*Myrcia fallax* (Rich.) DC. (Myrtaceae)	Native	X	X	X	X		X
*Myrcia guianensis* (Aubl.) DC. (Myrtaceae)	Native	X	X	X	X		X
*Myrsine guianensis* (Aubl.) Kuntze (Primulaceae)	Native	X	X	X	X	X	
*Nectandra megapotamica* (Spreng.) Mez (Lauraceae)	Native	X	X	X	X	X	X
*Ocimum basilicum* L. (Lamiaceae)	Cultivated	Occurrence: North, Northeast, Central-West, Southeast, and South					
*Ocimum canum* Sims (Lamiaceae)	Naturalized	X	X	X	X		
*Ocotea diospyrifolia* (Meisn.) Mez (Lauraceae)	Native			X	X	X	
*Passiflora foetida* L. (Passifloraceae)	Native	X	X	X	X	X	X
*Passiflora pohlii* Mast. (Passifloraceae)	Native			X	X		X
*Passiflora suberosa* L. (Passifloraceae)	Native	X	X	X	X		
*Pentaclethra macroloba* (Willd.) Kuntze (Fabaceae)	Native	X					
*Pereskia aculeata* Mill. (Cactaceae)	Native		X	X	X	X	
*Peritassa campestris* Cambess.) A.C. Sm. (Celastraceae)	Native			X	X		
*Persea americana* Mill. Var. americana (Lauraceae)	Naturalized				X		
*Peumus boldus* Molina (Monimiaceae)	Naturalized	X	X	X	X		X
*Piper augustum* Rudge (Piperaceae)	Native	X					
*Piper umbellatum* L. (Piperaceae)	Native	X		X	X		
*Piptadenia viridiflora* (Kunth) Benth. (Fabaceae)	Native		X	X			X
*Poincianella pyramidalis* (Tul.) L.P.Queiroz (Fabaceae)	Native		X				
*Porophyllum obscurum* (Spreng.) DC. (Asteraceae)	Native		X	X		X	
*Portulaca elatior* Mart. Ex Rohrb. (Portulacaceae)	Native		X	X	X		
*Pouteria ramiflora* (Mart.) Radlk. (Sapotaceae)	Native	X	X	X	X		
*Pouteria torta* (Mart.) Radlk. (Sapotaceae)	Native	X	X	X	X		
*Psidium guajava* L. (Myrtaceae)	Naturalized	X	X	X	X	X	
*Schinopsis brasiliensis* Engl. (Anacardiaceae)	Native		X	X			
*Rosmarinus officinalis* L. (Lamiaceae)	Cultived	Occurrence: North, Northeast, Central-West, Southeast, and South					
*Senna rugosa* (G.Don) H.S.Irwin & Barneby (Fabaceae)	Native	X	X	X	X		
*Serjania lethalis* A.St.-Hil. (Sapindaceae)	Native	X	X	X	X		X
*Sideroxylon obtusifolium* (Roem. & Schult.) T.D.Penn. (Sapotaceae)	Native		X	X	X		X
*Syzygium cumini* (L.) Skeels (Myrtaceae)	Naturalized	X		X	X		X
*Vernonia polysphaera* Baker (Asteraceae)	Native	X			X		
*Vitex cymosa* Bertero ex Spreng. (Lamiaceae)	Native	X	X	X	X		X
*Vochysia divergens* Pohl (Vochysiaceae)	Native	X		X			X
*Ximenia americana* L. (Ximeniaceae)	Native	X	X	X	X		
Attalea speciosa * Mart. Ex Spreng. (Arecaceae)	Native	X		X			
Buchenavia tetraphylla * (Aubl.) R.A.Howard (Combretaceae)	Native	X	X	X			
Caryocar coriaceum * Wittm. (Caryocaraceae)	Native			X			
Erythroxylum subrotundum * A.St.-Hil. (Erythroxylaceae)	Native		X	X	X		
Eugenia dysenterica * (Mart.) DC (Myrtaceae)	Native		X	X	X		
Harrisia adscendens * (Gürke) Britton & Rose (Cactaceae)	Native		X				
Lychnophora markgravii * G.M. Barroso (Asteraceae)	Native		X	X			
Miconia latecrenata * (DC.) Naudin (Melastomataceae)	Native				X		
Miconia willdenowii * Klotzsch ex Naudin (Melastomataceae)	Native				X		
Myrciaria pilosa * Sobral & Couto (Myrtaceae)	Native		X		X		
Ocotea minarum * (Nees & Mart.) Mez (Lauraceae)	Native			X	X		
Ocotea silvestris * Vattimo-Gil (Lauraceae)	Native				X	X	
Passiflora alata * Curtis (Passifloraceae)	Native	X		X	X	X	
Poincianella pyramidalis * (Tul.) L.P.Queiroz (Fabaceae)	Native		X				
*Psidium cattleianum* * Sabine (Myrtaceae)	Native		X	X	X		
Simaba ferruginea * A.St.-Hil. (Simaroubaceae)	Native			X			
Siparuna guianensis * Aubl. (Siparunaceae)	Native	X	X	X	X		X
Spondias tuberose * Arruda (Anacardiaceae)	Native		X	X			
Stryphnodendron adstringens * (Mart.) Coville (Fabaceae)	Native		X	X			
Vanillosmopsis arborea * (Gardner) Baker (Asteraceae)	Native		X	X			
Total		52	54	71	61	13	25

* Endemic plants in Brazil.

## Data Availability

Data are available upon request.
